# Letter to Editor about Comparison of accuracy, efficiency and safety between robotic-assisted and non-robotic-assisted deep brain stimulation: systematic review and/or meta-analysis

**DOI:** 10.1097/JS9.0000000000003694

**Published:** 2025-10-14

**Authors:** Zhi-Gang Cao

**Affiliations:** Department of Neurology, Sichuan Taikang Hospital, Chengdu, SC, China

*Dear Editor*,

We read with great interest the recent meta-analysis comparing robot-assisted deep brain stimulation (RA-DBS) with non-robot-assisted approaches^[1]^. The authors concluded that RA-DBS is comparable in terms of efficiency and safety, and may offer advantages in accuracy. This review provides a valuable synthesis of current evidence. However, we would like to highlight several dimensions that were not fully addressed, which make it difficult to interpret the “superiority” of robotic DBS over conventional DBS in a straightforward manner.

First, while accuracy was chosen as a primary outcome, its clinical significance is nuanced. A target point error difference of 0.3 mm may be statistically significant, yet unlikely to translate into noticeable changes in patient-reported outcomes or quality of life. Given that stimulation parameters can be adjusted postoperatively, the boundary between “technical precision” and “clinical effectiveness” becomes blurred. Considering that the two approaches showed no significant differences in other aspects (e.g., intracranial hemorrhage, complication rates, operative time, anesthesia duration), the key question may not be whether robotic DBS is more accurate, but whether greater accuracy consistently leads to patient-centered benefits.

Second, efficiency was assessed mainly through perioperative time, which risks overlooking broader system-level efficiency issues. Robotic platforms entail costs for acquisition, maintenance, and personnel training. These additional demands on time and resources were not accounted for in the pooled analysis. What appears as “comparable” intraoperative performance may, in reality, reflect shifts in institutional workload and redistribution of expertise.

Third, the comparator in these analyses, frame-based and frameless systems, has long been regarded as the clinical gold standard^[2]^. By contrast, robotic platforms are rapidly evolving. The true value of robotics may lie less in outperforming existing frame systems and more in enabling workflows that frames cannot easily support, such as seamless integration with advanced imaging or minimally invasive targeting of novel brain regions. In this sense, robotics may be better viewed as a platform for surgical innovation rather than merely an alternative tool.

Finally, the meta-analysis underscores a paradox: the heterogeneity of robotic systems, study designs, and outcome definitions complicates pooled conclusions, yet such heterogeneity mirrors the diversity of real-world surgical practice. Instead of seeking a single summary estimate of accuracy or efficiency, it may be more meaningful to identify the conditions under which RA-DBS offers the greatest value – for instance, during early adoption in low-volume centers, or in high-volume teams with established frame-based expertise.

In conclusion, while this study provides valuable quantitative comparisons, we believe the more pressing challenge is to redefine the dimensions by which robotic neurosurgery is evaluated. Rather than asking whether RA-DBS is “better” than non-robotic DBS, the focus should shift toward understanding in which patient groups and under what conditions robotic systems can achieve broader clinical goals and demonstrate their unique advantages. We declare that the writing of this article complies with the TITAN guidelines^[3]^.

## Data Availability

No datasets were generated or analyzed for this study.
